# Supplementary material for: Associations between Traffic-Related Air Pollution and Cognitive Function in Australian Urban Settings: The Moderating Role of Diabetes Status
Source: Toxics. 2022 May 27;10(6):289. doi: 10.3390/toxics10060289 (PMC9228131; doi:10.3390/toxics10060289)
Supplement: Supplementary file 1 [file toxics-10-00289-s001.zip › toxics-1704544-supplementary.pdf]

Communication

Associations between traffic-related air pollution and cognitive function in Australian urban settings: the moderating role of diabetes status

Supplementary Material

Table of Contents

Scheme 1. Detailed description of covariates.

Scheme 2. Analysis of missing data.

Figure S1. Directed acyclic graph (DAG) depicting the hypothesised relations between road density (a traffic-related air pollution proxy) and cognitive function. Through the DAG, we identified the confounders (pink circles) to be included in the regression models.

Table S1. Outline of regression analyses: Estimation of total effects of traffic-related air pollution (TRAP) measures on cognitive function.

Table S2. Descriptive statistics of neighbourhood environment measures (M ± SD).

Table S3. Associations of traffic-related air pollution (TRAP) measures with cognitive function (complete case analyses; n = 3,261).

Table S4. Moderation effects of diabetes status on the associations between traffic-related air pollution (TRAP) measures with cognitive function (complete case analyses; n = 3,261).

Section S1. Detailed description of covariates

Self-reported covariates included age (continuous); sex (male/female); household income (categorized: Up to $49,999, $50,000–$99,999 and ≥ $100,000); area-level socio-economic status (Index of Relative Socio-economic Advantage and Disadvantage (IRSAD) [1], educational attainment (Primary or secondary school; Trade/Technician; Associate degree/Diploma/Nursing/teaching; Bachelor degree or postgraduate diploma and higher), employment status (Not employed/Paid employment/Volunteer), English-speaking background (yes/no), living arrangement (Couple with children/Couple without children/Other).

To account for the confounding effects of residential self-selection, that is, people selecting to live in areas that provide the facilities that satisfy their preferred lifestyle [2], two variables based on participants' responses to 5-point-scale items assessing the importance of reasons for choosing to live in the current neighborhood [3] were included: one related to access to recreational facilities (1 item), the other related to good access to various destinations (4 items).

Population density was defined as the number of persons per hectare and was derived using Australian Bureau of Statistics (ABS) mesh block data

from the 2011 Census [4]. Density of greenspace was calculated using the normalized difference vegetation index (NDVI). This index has values ranging from −1 to 1. Lower values (< 0.1) indicate bare/rocky ground, moderate values (0.2–0.3) represent shrubs and grassland, and high values (0.6–0.8) represent densely vegetated areas. Infrared and red band NASA Landsat (LANDSAT5 and 7) satellite images sourced from Earth Explore from September 2011 to March 2012, depending on availability and with < 10% cloud cover, were used to calculate NDVI. The percentage of buffer areas taken up by commercial and industrial land use was derived from the main planned land use for mesh blocks reported in the ABS mesh block data from 2011[5]. A land use mix score, ranging from 0 to 1, related to outdoor physical activity was computed to quantify the heterogeneity of residential land, parkland and bluespace (water bodies), and was calculated using the same ABS mesh block data [5].

Section S2. Analysis of missing data

Over 21% of cases had missing data on at least one variable. Predictors of missingness (the odds of having incomplete data on any of the examined variables) were determined using generalized linear mixed models with binomial variance and logit link functions and random intercepts at the Statistical Area 1 (SA1) level. The odds of having missing data were higher in older participants ($p < 0.001$), those of non-English speaking background ($p < 0.001$), with lower household income ($p = 0.012$), not working or volunteering ($p = 0.009$), with IGT/IFG or diabetes (p < 0 .001), living in areas with a lower Index of Relative Advantage and Disadvantage (IRSAD) ($p = 0.011$) and with lower scores on the memory test ($p = 0.022$). Missingness was also more prevalent in people for whom access to services was an important reason for living in their neighbourhood ($p = 0.042$) and those living in areas with higher population density ($p < 0.001$) and lower street intersection density ($p = 0.007$). As data were at least missing at random (MAR) rather than missing completely at random (MCAR), ten imputed datasets were created for the regression analyses as recommended by Rubin[6] and Van Buuren [7]. Multiple imputations by chained equations were performed following currently recommended model-building and diagnostic procedures [8] and using the package 'mice' [9] in Rversion 4.0.0.

Figure S1. Directed acyclic graph (DAG) depicting the hypothesised relations between road density (a traffic-related air pollution proxy) and cognitive function. Through the DAG, we identified the confounders (pink circles) to be included in the regression models.

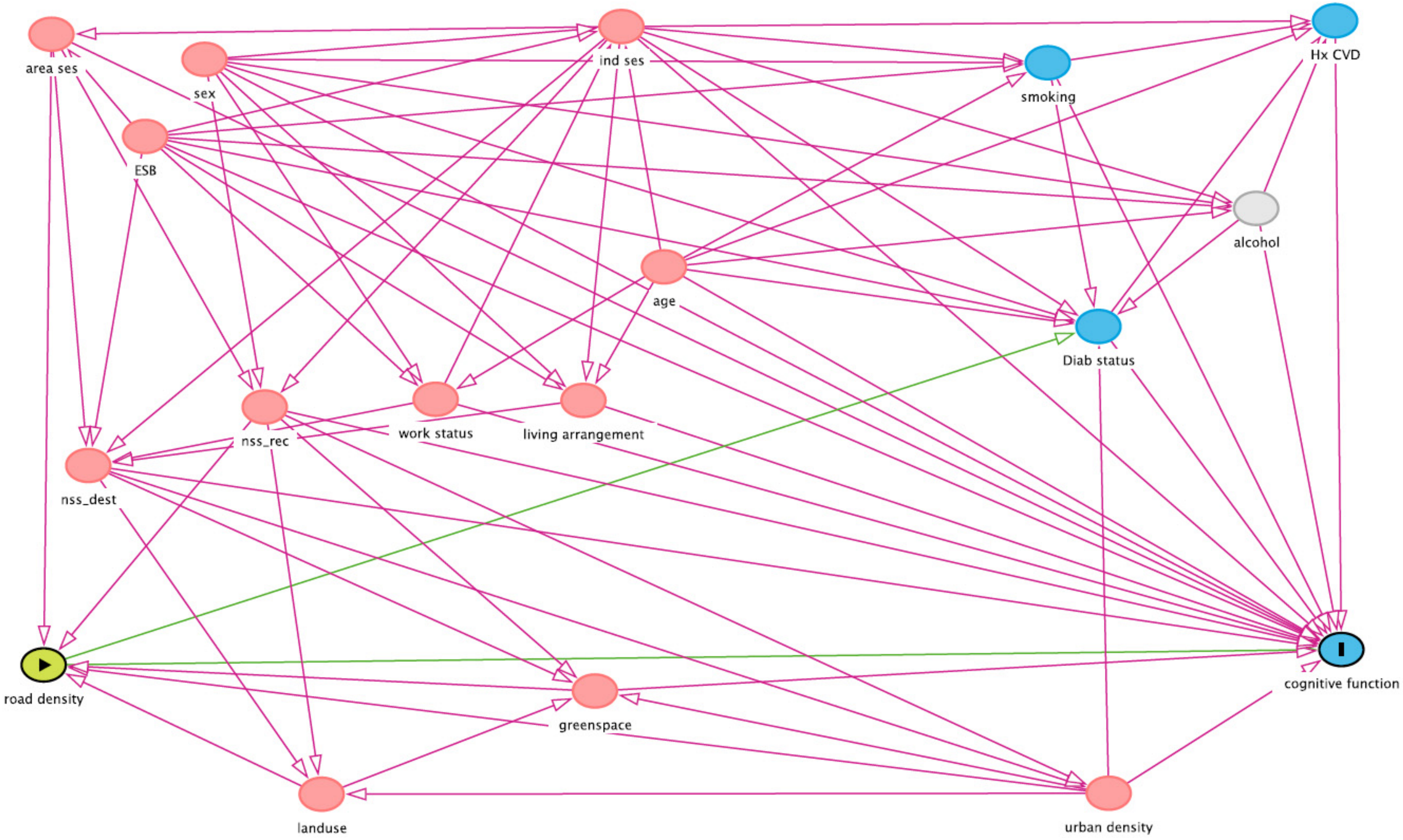

.

toxics MDPI

Table S1. Outline of regression analyses: Estimation of total effects of traffic-related air pollution (TRAP) measures on cognitive function

Models	Exposure(s) / Effect(s)	Covariates	Regression Models	Results Table
1TE	Road density (100 m/km²)	Age, sex, English-speaking background, living arrangements, educational attainment, household income, work status, area-level IRSAD, dwelling density, commercial land use (%), industrial land use (%), mean NDVI, land use mix, residential self-selection	Separate sets of GAMMs for each cognitive function: GAMMs with a linear term and GAMMs with a smooth curvilinear term for each TRAP measure. GAMMs with Gaussian variance and identity link functions.	Complete case analyses = Table S3 Multiple imputations analyses = Table 3
2TE	Minor and major road densities (100 m/km²)	As above	As above	
3TE	Distance to nearest busy road (100 m)	As above	As above	
1MD	Diabetes status as moderator of effect of road density	As above + diabetes status as an interaction covariate	As above	Complete case analyses = Table S4 Multiple imputation analyses = Table 4
2MD	Diabetes status as moderator of effect of minor and major road densities (100 m/km²)	As above	As above	
3MD	Diabetes status as moderator of effect of distance to nearest busy road (100 m)	As above	As above	

Notes. TE, total-effect models; MD, models examining the moderating effects of diabetes status on TRAP-cognition associations; GAMMS, generalised linear mixed models; IRSAD, Index of Relative Advantage and Disadvantage; NDVI, Normalised Difference Vegetation Index.

Table S2. Descriptive statistics of neighbourhood environment measures (M ± SD).

Characteristic	Total sample (*n* = 4141)	Diabetes status		
		Diabetes (*n* = 405)	IGT/ IFG (*n* = 620)	Normal glucose tolerance (*n* = 3,003)
Dwelling density (dwellings/ha)				
200 m Euclidean buffer	2.92 ± 5.33	3.04 ± 5.07	2.85 ± 6.24	2.86 ± 5.06
300 m Euclidean buffer	2.87 ± 4.85	2.99 ± 4.89	2.75 ± 5.58	2.82 ± 4.59
500 m Euclidean buffer	2.75 ± 4.44	2.84 ± 4.19	2.63 ± 5.19	2.72 ± 4.24
1000 m Euclidean buffer	2.31 ± 3.60	2.38 ± 3.08	2.24 ± 3.95	2.28 ± 3.52
1600 m Euclidean buffer	2.40 ± 2.90	2.46 ± 2.53	2.36 ± 2.99	2.38 ± 2.91
Commercial land use (%)				
200 m Euclidean buffer	1.32 ± 6.58	1.28 ± 6.13	1.25 ± 5.89	1.34 ± 6.79

300 m Euclidean buffer	1.68 ± 6.35	1.63 ± 5.66	1.58 ± 5.61	1.69 ± 6.59
500 m Euclidean buffer	2.15 ± 5.94	2.21 ± 5.18	1.96 ± 5.26	2.17 ± 6.18
1000 m Euclidean buffer	2.51 ± 5.14	2.72 ± 4.55	2.59 ± 4.95	2.44 ± 5.27
1600 m Euclidean buffer	2.58 ± 4.31	2.75 ± 3.85	2.76 ± 4.30	2.49 ± 4.36
Industrial land use (%)				
200 m Euclidean buffer	0.47 ± 4.35	0.78 ± 6.76	0.43 ± 4.82	0.44 ± 3.88
300 m Euclidean buffer	0.70 ± 4.63	0.81 ± 6.01	0.60 ± 4.59	0.72 ± 4.48
500 m Euclidean buffer	1.14 ± 4.94	1.17 ± 5.00	1.11 ± 4.99	1.15 ± 4.98
1000 m Euclidean buffer	2.14 ± 5.09	2.01 ± 4.58	2.37 ± 5.37	2.11 ± 5.11
1600 m Euclidean buffer	2.86 ± 4.98	2.87 ± 4.74	3.18 ± 5.01	2.78 ± 4.97
Land use mix – entropy score*				
200 m Euclidean buffer	0.24 ± 0.22	0.23 ± 0.22	0.23 ± 0.22	0.24 ± 0.22
300 m Euclidean buffer	0.31 ± 0.23	0.31 ± 0.23	0.30 ± 0.23	0.32 ± 0.23
500 m Euclidean buffer	0.41 ± 0.21	0.41 ± 0.21	0.40 ± 0.21	0.41 ± 0.21
1000 m Euclidean buffer	0.52 ± 0.17	0.52 ± 0.17	0.52 ± 0.17	0.52 ± 0.17
1600 m Euclidean buffer	0.57 ± 0.14	0.57 ± 0.13	0.58 ± 0.13	0.57 ± 0.14
Normalised Difference Vegetation Index				
200 m Euclidean buffer	0.34 ± 0.11	0.33 ± 0.11	0.32 ± 0.11	0.34 ± 0.11
300 m Euclidean buffer	0.34 ± 0.11	0.33 ± 0.11	0.32 ± 0.11	0.34 ± 0.11
500 m Euclidean buffer	0.34 ± 0.11	0.33 ± 0.11	0.33 ± 0.11	0.34 ± 0.11
1000 m Euclidean buffer	0.34 ± 0.11	0.33 ± 0.11	0.33 ± 0.11	0.34 ± 0.11
1600 m Euclidean buffer	0.34 ± 0.11	0.33 ± 0.10	0.33 ± 0.10	0.35 ± 0.11

Notes. M, mean; SD, standard deviation; IGT, impaired glucose tolerance; IFG, impaired fasting glucose; ha, hectare; * includes: residential land use, parkland, blue space and other land uses (excluding commercial and industrial land use) - ranges from 0 to 1 (0 = single land use; 1 = maximal land use heterogeneity).

Table S3. Associations of traffic-related air pollution (TRAP) measures with cognitive function (complete case analyses; n = 3,261).

TRAP measures	Memory (CVLT score)			Processing speed (SDMT score)		
	β	95% CI	p	β	95% CI	p
Road density (100 m / km^2)						
200 m Euclidean buffer	**0.003**	**0.001, 0.006**	**0.001**	0.007	−0.001, 0.015	0.101
300 m Euclidean buffer	**0.003**	**0.0002, 0.005**	**0.030**	0.005	−0.004, 0.013	0.310
500 m Euclidean buffer	**0.003**	**0.001, 0.006**	**0.013**	0.005	−0.005, 0.015	0.315
1000 m Euclidean buffer	**0.004**	**0.001, 0.007**	**0.005**	0.006	−0.005, 0.018	0.279
1600 m Euclidean buffer	**0.005**	**0.001, 0.007**	**0.005**	***0.011***	***−0.001, 0.023***	***0.083***
Minor road density (100 m / km^2)						
200 m Euclidean buffer	**0.003**	**0.003, 0.005**	**0.027**	0.003	−0.006, 0.012	0.543
300 m Euclidean buffer	0.002	−0.001, 0.005	0.143	0.003	−0.008, 0.013	0.661
500 m Euclidean buffer	0.002	−0.001, 0.006	0.185	−0.003	−0.026, 0.010	0.628
1000 m Euclidean buffer	0.003	−0.002, 0.006	0.224	−0.004	−0.019, 0.011	0.617
1600 m Euclidean buffer	0.002	−0.002, 0.007	0.364	−0.0003	−0.018, 0.017	0.977
Major road density (100 m / km^2)						
200 m Euclidean buffer	0.002	−0.002, 0.007	0.321	0.007	−0.012, 0.026	0.497
300 m Euclidean buffer	0.001	−0.004, 0.006	0.707	−0.003	−0.022, 0.017	0.775
500 m Euclidean buffer	0.004	−0.002, 0.011	0.203	0.006	−0.020, 0.031	0.667
1000 m Euclidean buffer	***0.010***	***−0.0002, 0.021***	***0.055***	0.016	−0.024, 0.056	0.436
1600 m Euclidean buffer	**0.015**	**0.002, 0.029**	**0.024**	0.037	−0.014, 0.088	0.153
Distance to nearest busy road (100 m)	−0.003	−0.021, 0.015	0.729	−0.038	−0.038, -0.106	0.267

Notes. *β*, regression coefficient; CI, confidence intervals; *p*, *p*-value; CVLT, California Verbal Learning Test; SDMT, Symbol-Digit Modality Test. Estimates of regression coefficient adjusted for covariates listed in Table S1. In bold are statistically significant associations at a probability level of 0.05. In bold italics are statistically significant associations at a probability level of 0.10.

Interpretation of results in Table S3:

Positive associations were observed between road density and memory irrespective of the residential buffer size, while only road density within 1.6 km from the participants' homes showed a weak (marginal) positive association with processing speed. Minor road density within 200 m buffers, and major road density within larger buffer sizes, were positively related to memory. No other significant associations were observed.

Table S4. Moderation effects of diabetes status on the associations between traffic-related air pollution (TRAP) measures with cognitive function (complete case analyses; *n* = 3,261).

TRAP measures	Memory (CVLT score)		Processing speed (SDMT score)	
	***F* (2, 3238)**	***p***	***F* (2, 3238)**	***p***
Road density (100 m / km^2)				
200 m Euclidean buffer	1.22	0.296	0.93	0.395
300 m Euclidean buffer	1.23	0.293	0.52	0.594
500 m Euclidean buffer	0.57	0.564	0.14	0.872
1000 m Euclidean buffer	1.56	0.314	0.50	0.609
1600 m Euclidean buffer	1.27	0.282	0.57	0.566
Minor road density (100 m / km^2)				
200 m Euclidean buffer	1.33	0.265	0.31	0.733
300 m Euclidean buffer	0.45	0.639	0.37	0.693
500 m Euclidean buffer	0.73	0.483	0.42	0.658
1000 m Euclidean buffer	0.28	0.755	1.56	0.208
1600 m Euclidean buffer	0.45	0.640	***2.58***	***0.076***
Major road density (100 m / km^2)				
200 m Euclidean buffer	0.65	0.522	0.15	0.861
300 m Euclidean buffer	***2.45***	***0.086***	1.11	0.329
500 m Euclidean buffer	0.91	0.402	0.52	0.594
1000 m Euclidean buffer	0.77	0.462	0.51	0.599
1600 m Euclidean buffer	1.65	0.192	2.16	0.116
Distance to nearest busy road (100 m)	0.74	0.477	0.31	0.737

Notes. F, F-ratio; *p*, *p*-value; CVLT, California Verbal Learning Test; SDMT, Symbol-Digit Modality Test. Estimates of regression coefficient adjusted for covariates listed in Table S1. In bold italics are statistically significant moderation effects of diabetes status on TRAP-cognitive function at a probability level of 0.10.

The F-ratio values reported in this table represent tests of significance of the overall moderating effect of diabetes status on the relationships between TRAP measures and cognitive function. The moderating effects were represented by two regression coefficients: one estimating the difference in TRAP-cognition associations between normal glucose tolerance controls and participants with IGT/IFG; the other estimating the difference in TRAP-cognition associations between normal glucose tolerance controls and those with diabetes. The F-ratio tested the overall significance of both regression coefficients.

Interpretation of results in Table S4

In general, the data did not support the presence of a moderating effect of diabetes status on the associations between TRAP measures and cognitive function.

References for Supplementary Material

29. Rubin, D.B. *Multiple imputation for non-response in surveys*; Wiley & Sons: New York, 1987.
30. van Buuren, S. *Flexible imputation of missing data*, 2nd Edition ed.; Chapman & Hall: Boca Raton, Florida, 2018.
31. van Buuren, S.; Groothuis-Oudshoorn, K. mice: Multivariate Imputation by Chained Equations in R. *J. Stat. Softw* **2011**, 1-67.
32. R Core Team *R: A language and environment for statistical computing*, https://www.R-project.org/: Vienna, Austria, 2021.
48. Lamb, K.E.; Thornton, L.E.; King, T.L.; Ball, K.; White, S.R.; Bentley, R.; Coffee, N.T.; Daniel, M. Methods for accounting for neighbourhood self-selection in physical activity and dietary behaviour research: a systematic review. *International Journal of Behavioral Nutrition and Physical Activity* **2020**, *17*, 45, doi:10.1186/s12966-020-00947-2.
49. Cerin, E.; Leslie, E.; Toit, L.d.; Owen, N.; Frank, L.D. Destinations that matter: Associations with walking for transport. *Health & Place* **2007**, *13*, 713-724, doi:https://doi.org/10.1016/j.healthplace.2006.11.002.
50. Australian Bureau of Statistics (ABS). *Census of population and housing: mesh block counts, 2011 (cat. no. 2074)*; Canberra, Australia, 2011.
51. Australian Bureau of Statistics (ABS). *Australian statistical geography Standard (ASGS) Volume 1 - Main Structure and Greater capital City Statistical Areas (Cat No. 1270.0.55.001)*; Australian Bureau of Statistics: Canberra, Australia, 2011.
52. Australian Bureau of Statistics (ABS). 2033.0.55.001 - Census of Population and Housing: Socio-Economic Indexes for Areas (SEIFA), Australia, 2011. Available online: (accessed on 24 March 2022).
